# Transarterial chemoembolization combined with metformin improves the prognosis of hepatocellular carcinoma patients with type 2 diabetes

**DOI:** 10.3389/fendo.2022.996228

**Published:** 2022-09-15

**Authors:** Miao-Ling Chen, Chun-Xue Wu, Jian-Bo Zhang, Hao Zhang, Yuan-Dong Sun, Shi-Lin Tian, Jian-Jun Han

**Affiliations:** ^1^ Shandong Cancer Hospital and Institute, Shandong First Medical University and Shandong Academy of Medical Sciences, Jinan, China; ^2^ Interventional Medicine Center, Shandong Cancer Hospital and Institute Affiliated Shandong First Medical University and Shandong Academy of Medical Sciences, Jinan, China; ^3^ Pathology Department, Shandong Cancer Hospital and Institute Affiliated Shandong First Medical University and Shandong Academy of Medical Sciences, Jinan, China

**Keywords:** metformin, hepatocellular carcinoma, transarterial chem-embolization, type 2 diabetes mellitus, prognosis

## Abstract

**Objective:**

The study aims to investigate the effect of metformin on Hepatocellular carcinoma (HCC) patients with type 2 diabetes mellitus (T2DM) who received transarterial chemoembolization (TACE) for the first time.

**Methods:**

From January 2016 to December 2019, T2DM patients diagnosed with HCC in Shandong Cancer Hospital and treated with TACE were included in this retrospective study. Overall survival (OS) and Progression-free survival (PFS) were compared between patients treated with metformin and other antidiabetics. Univariate and multivariate Cox regression models were used to evaluate the independent risk factors associated with OS and PFS. And sub-analysis was performed to investigate whether metformin could give a survival advantage in each Barcelona Clinic Liver Cancer (BCLC) stage of HCC. Propensity score matched (PSM) analyses based on patient and tumor characteristics were also conducted.

**Results:**

A total of 123 HCC patients with T2DM underwent TACE, of which 50 (40.65%) received treatment with metformin. For the whole cohort, the median OS (42 vs 32 months, p=0.054) and PFS (12 vs 7 months, P=0.0016) were longer in the metformin group than that in the non-metformin group. Multi-analysis revealed that BCLC stage, BMI (Body Mass Index), and metformin use were independent predictors of OS. Metformin use was independently associated with recurrence. After PSM, 39 matched pairs were identified. The use of metformin was associated with a numerically longer m OS (43 vs 35 months, P=0.183) than the use of other anti-diabetics. And the difference in median PFS (13 vs 7 months, p=0.018) between the metformin group and non-metformin group remained significant.

**Conclusion:**

The combination of transarterial chemoembolization and metformin may be associated with better OS and PFS in HCC patients with T2DM.

## Introduction

In China, liver cancer ranks third among the cancer-related causes of death (1). Potentially curative treatments for Hepatocellular carcinoma (HCC) include surgery (resection or transplant), radiofrequency ablation (RFA), and percutaneous ethanol injection (PEI). However, approximately 25-70% of patients with HCC are diagnosed with mediate and advanced HCC, which is regarded as incurable (2). One of the most common treatment modalities for these patients is transarterial chem-embolization (TACE).

TACE, the first-line treatment modality for Barcelona Clinic Liver Cancer (BCLC) stage B HCC according to the BCLC staging system, considerably improves the survival of patients with unresectable HCC (3). A study by Zhang et al. shows that TACE achieves comparable long-term survival results to liver resection in HCC patients with BCLC stage A. But in cases with tumor diameters <= 6 cm, the TACE group didn’t show a survival benefit compared with the liver resection group (4). The 5-year overall survival (OS) for TACE ranges from 0% to 74.2% due to the clinical characteristics of patients varying between studies (5–7). Among HCC patients who received TACE, poor long-term survival is found by several studies to correlate with tumor recurrence (the primary cause of poor prognosis), BCLC stage, diabetes, liver cirrhosis, tumor size, and tumor number.

Obesity and type 2 diabetes mellitus (T2DM) have recently become increasingly prevalent and have been reported to be related to increased morbidity and mortality from many cancers, particularly HCC (8, 9). Patients with diabetes have a 126% higher risk of cancer-related death from liver cancer than those without diabetes (10). At the same time, obesity and diabetes-induced insulin resistance are one of the main triggers for the development and progression of Nonalcoholic Fatty Liver Disease (NAFLD). Metformin, as an insulin sensitizer, may improve insulin resistance (11). Through this way of action, it is possible to reduce the development of NADSH, cirrhosis, and HCC (12). Moreover, it is suggested that metformin use might have antitumor effects against HCC in specific treatments, including radiofrequency ablation (RFA) and hepatectomy.

Since there are no relevant studies on the effect of metformin on TACE in HCC patients with T2DM, we conducted a retrospective analysis on these patients to determine whether metformin can reduce tumor recurrence and increase the survival of patients with TACE in different BCLC stages.

## Material and methods

### Patients

From April 2016 to December 2019, a total of 123 HCC patients with T2DM who received TACE at Shandong Tumor Hospital were included in this research. Among them, 50 patients were treated with metformin, and 73 patients were treated with other antidiabetics. The diagnosis of HCC is based on the Guidelines for the Diagnosis and Treatment of Hepatocellular Carcinoma (China, 2019 Edition) by a liver biopsy or imaging examination, including contrast-enhanced computed tomography (CE-CT) or magnetic resonance (CE-MRI). HCCs were staged using the BCLC (Barcelona Clinic Liver Cancer) staging system one month before the TACE. The inclusion criteria were as follows: 1) 18-75 years old; 2) metformin-treated for more than three months before the TACE; 3) Child-Pugh A-B; 4) Eastern Cooperative Oncology Group (ECOG) score 0-1; 5) no more than five and at least one detectable lesion; 6) without other malignant tumors within five years. Patients were eliminated if they had any of the following: 1) a history of other cancers; 2) a life expectancy of fewer than three months; or 3) no baseline data. The medical histories, laboratory tests, and imaging examinations of patients were documented.

Shandong Cancer Hospital ethics committee (Shandong, China) approved the project. Due to the retrospective nature of this investigation, the permission of patients for inclusion was waived.

### TACE procedure

Under the guidance of Digital Subtraction Angiography (DSA), the Seldinger method was used to puncture the right femoral artery. After the 5F catheter was successfully inserted into the hepatic artery, a diagnostic hepatic angiography was performed to assess the location, size, number, and feeding arteries of the tumor. The catheter is selectively positioned in the artery feeding the tumor. The mixed emulsion of chemotherapeutic drugs and lipiodol is delivered to the target tumor through the catheter, and then standardized gelatin sponge particles are used to embolize the blood vessels supplying the tumor. Finally, the catheter and the artery sheath were removed. The absence of tumor stain in dynamic DSA images was the requirement for a successful procedure.

All patients received the same regimen of TACE, which comprised of the following drugs: Epirubicin (30-40 mg), oxaliplatin (100-150 mg), and 5-fluorouracil (2-2.5 g). The dosage of the drug is adjusted by clinical experts according to the patient’s laboratory and imaging test results and the patient’s actual physical condition. TACE was repeated as needed when the previous treatment was effective, tumor burden was reduced, the Alpha-fetoprotein (AFP) level was still high or elevated, and the angiography showed that there were still lesions without lipiodol or new lesions, normal or mildly abnormal liver function.

### Drug exposure and group

Antidiabetic exposure was documented in the Clinical Computer-Based Patient Record (CPR) at Shandong Tumor Hospital. Patients treated with metformin for more than 3 months were categorized as “metformin users”, regardless of whether they received other antidiabetics (including Insulin, Thiazolidinediones, Sulfonylureas, and so on). Patients took metformin at a dose of 2 grams a day. The dose-dependent effect of metformin was analyzed according to the different cumulative duration of metformin use before TACE. Patients who used 0.4 grams of sorafenib twice a day for more than four weeks were defined as “sorafenib users”. The drugs were administered until the disease progression, intolerable toxicity, or death. According to the BCLC staging system, the subgroup analysis was performed on HCC patients with T2DM who were treated with metformin or not.

### Follow-up and assessment

Each follow-up appointment involved medical history, laboratory testing, and imaging assessment. All patients were followed up after the first month of TACE, then every two to three months. OS was the objective endpoint of the study, respectively. PFS and tumor response was the secondary endpoints of the study. OS was defined as the interval between the first TACE procedure and either death or the last follow-up (considered censored). PFS was defined as the period between the initial treatment and radiologically confirmed recurrence. Tumor response rates included objective response rate (ORR) and disease control rate (DCR). ORR was defined as the percentage of complete response (CR) and partial response (PR) which was maintained for at least 4 weeks from the first radiological confirmation. DCR was defined as the ORR plus the percentage of patients with stable disease (SD). Tumor response was determined according to the modified Response Evaluation Criteria in Solid Tumors (m RECIST). Our study was censored on April 7, 2022.

### Statistical analysis

Normally distributed and skewed continuous variables were compared using Student’s t-test and Mann-Whitney U test, respectively, described as mean ± standard deviation (SD). The categorical variable was described as proportion using Pearson’s X^2^ test or Fisher’s exact test to compare.

Propensity score matching (PSM) was performed to minimize the selection bias. A propensity score was calculated for every patient by logistic regression based on significantly different and prognostic baseline variables. To construct a matched cohort, patients treated with metformin were matched 1:1 to patients treated without metformin by using the nearest-neighbor matching algorithm with the maximum allowed difference of 2% for propensity scores. Multivariate analysis using a Cox regression model was performed to define significant prognostic factors. Survival curves were estimated using the Kaplan-Meier method, and the differences in survival rates between groups were compared using the Log-rank test. For all tests, a P-value < 0.05 was considered statistically significant. Data analysis and graphing were performed using SPSS Statistic (version 26.0, USA) and R language version 4.1.3 (http://www.r-project.org/).

## Results

### Patient characteristics

A total of 123 HCC patients with T2DM underwent TACE for the first time during the study period, 50 patients received metformin, and 73 patients received other antidiabetics. The recorded clinical characteristics are shown in **Table 1**. 57.72% of patients were under the age of 60 and 108 (87.8%) patients were male. The most common cause of HCC is hepatitis B (79.67%), followed by hepatitis C (5.69%), alcohol (4.88%), NAFLD (1.63%), and others (8.13%). At the time of diagnosis, 97 (78.86%) patients had cirrhosis and almost 89 (72.36%) patients had overweight (body weight index, BMI >= 24 kg/m^2^). The BCLC stage of patients were as follows: stage A, 34.96%(n=43); stage B, 31.71%(n=39); stage C, 33.33%(n=41). A total of 6 patients treated with metformin had previously undergone hepatectomy with a median interval of 35 (26.652) months, and 1 patient treated with other antidiabetics with an interval of 4 months, p=0.0354. Patients treated with metformin were more likely to have multiple tumors (72.00% *vs* 52.05%, p=0.0422). Other clinical characteristics between the two groups were well balanced. Although statistically insignificant, the metformin group had a higher proportion of ORR (72.00% *vs* 58.9%, p= 0.1947) and DCR (98% *vs* 89.04%, p=0.1281) in the metformin group than in the non-metformin group.

**Table 1:** Clinical characteristics between the metformin group and the non-metformin group in the whole cohort.

Characteristic	Overall	Non-metformin Group	Metformin Group	*P-*Value
	n=123	n=73	n=50	
Age (%)				0.2116
<=60 years	71 (57.72)	46 (63.01)	25 (50.00)	
>60 years	52 (42.28)	27 (36.99)	25 (50.00)	
Gender (%)				1.0000
Female	15 (12.20)	9 (12.33)	6 (12.00)	
Male	108 (87.80)	64 (87.67)	44 (88.00)	
Etiology (%)				0.6724
HBV	98 (79.67)	57 (78.08)	41 (82.00)	
HCV	7 (5.69)	5 (6.85)	2 (4.00)	
NAFLD	2 (1.63)	1 (1.37)	1 (2.00)	
Alcohol	6 (4.88)	5 (6.85)	1 (2.00)	
others	10 (8.13)	5 (6.85)	5 (10.00)	
BMI (%)				0.1211
<24 kg/m2	34 (27.64)	24 (32.88)	10 (20.00)	
24-27.9 kg/m2	64 (52.03)	38 (52.05)	26 (52.00)	
≥28 kg/m2	25 (20.33)	11 (15.07)	14 (28.00)	
Child-Pugh Score (%)				0.9982
A	107 (86.99)	63 (86.30)	44 (88.00)	
B	16 (13.01)	10 (13.70)	6 (12.00)	
BCLC stage (%)				0.9823
A	43 (34.96)	26 (35.62)	17 (34.00)	
B	39 (31.71)	23 (31.51)	16 (32.00)	
C	41 (33.33)	24 (32.88)	17 (34.00)	
Liver cirrhosis (%)				0.3522
Absent	26 (21.14)	18 (24.66)	8 (16.00)	
Present	97 (78.86)	55 (75.34)	42 (84.00)	
Size (%)				0.7747
<=5 cm	51 (41.46)	29 (39.73)	22 (44.00)	
>5 cm	72 (58.54)	44 (60.27)	28 (56.00)	
Tumor number (%)				0.0422
single	49 (39.84)	35 (47.95)	14 (28.00)	
multiple	74 (60.16)	38 (52.05)	36 (72.00)	
FBG (%)				0.1198
<7 mmol/L	31 (25.20)	23 (31.51)	8 (16.00)	
7.0-7.6 mmol/L	17 (13.82)	8 (10.96)	9 (18.00)	
≥7.7 mmol/L	75 (60.98)	42 (57.53)	33 (66.00)	
T2DM duration (months) †	54.000 [21.500, 125.000]	54.000 [19.000, 125.000]	55.000 [27.500, 126.250]	0.6860
Hepatectomy before TACE (%)	7 (5.69)	1 (1.37)	6 (12.00)	0.0354
ALT (U/L)	32.300 [20.750, 51.150]	29.000 [20.000, 51.300]	34.100 [21.100, 50.075]	0.7909
AST (U/L)	36.700 [24.650, 56.300]	36.400 [25.800, 55.900]	36.850 [23.775, 57.750]	0.8248
Times of TACE	4.000 [2.000, 8.000]	4.000 [2.000, 8.000]	4.000 [3.000, 7.000]	0.9897
Sorafenib after TACE (%)	38 (30.89)	22 (30.14)	16 (32.00)	0.9832
Treatment after TACE (%) ‡	20 (16.26)	11 (15.07)	9 (18.00)	0.8540
Tumor response (%)				0.2376
CR	9 (7.32)	5 (6.85)	4 (8.00)	
PR	70 (56.91)	38 (52.05)	32 (64.00)	
SD	35 (28.46)	22 (30.14)	13 (26.00)	
PD	9 (7.32)	8 (10.96)	1 (2.00)	
ORR (%) §	114 (92.68)	65 (89.04)	49 (98.00)	0.1281
DCR (%) ¶	79 (64.23)	43 (58.90)	36 (72.00)	0.1947

Data are shown as numbers of events with percentages in parentheses or median [interquartile range, IQR]. HBV, hepatitis B virus; HCV, hepatitis C virus; NAFLD, nonalcoholic fatty liver disease; BMI, body mass index; ECOG, Eastern Cooperative Oncology Group; BCLC, Barcelona Clinic Liver Cancer; T2DM, type 2 diabetic mellitus; FBG, Fasting blood sugar; ALT, Alanine aminotransferase; AST, Aspartate aminotransferase; TACE, transarterial chemoembolization; CR, complete response; PR, partial response; SD, stable disease; PD, progressive disease; ORR, objective response rate; DCR, disease control rate

†: T2DM duration before the first TACE

‡: Including radiofrequency, microwave ablation and Hepatectomy.

§: Sum of CR and PR.

¶: Sum of CR, PR, and SD.

### Survival outcome

**Figure 1:**
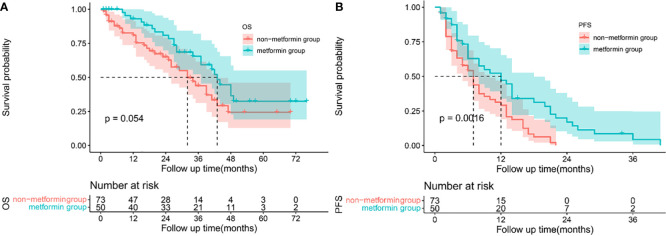
Survival analysis: Kaplan-Meier plots comparing metformin users versus non-metformin users among HCC patients with T2DM undergoing TACE. Overall survival **(A)** and progression-free survival **(B)** curves in the whole cohort.

Patients treated with metformin and other antidiabetic medications had a median follow-up of 35.5 months and 14.5 months, respectively. Patients treated with metformin had a better OS than that treated with other antidiabetics, as shown in **Figure 1A**. The cumulative OS rates at 1, 2, and 3 years for patients treated with metformin were better than patients treated with other antidiabetics (1-year, 93.02% vs 80.87%; 2-year 80.90% vs 65.00%; 3-year 65.49% vs 43.84%). The median OS of patients treated with metformin and other antidiabetics was 42 months (95% CI 38.430-47.570) and 32 months (95% CI 22.540-41.460), respectively (p=0.054). Treated with metformin in HCC patients with T2DM was associated with lower tumor recurrence compared with treatment with other antidiabetics (1 year 52.39% vs 29.19%; 2-year 17.03% vs 0.00%; 3-year 4.26% vs 0.00%), **Figure 1B**. The median PFS of patients treated with metformin and other antidiabetics was 12 months (95% CI 6.093-17.907) and 7 months (95% CI 5.132-8.868), respectively (P=0.0016).

### Univariate and multivariate analysis for overall survival and progression-free survival

Significant prognostic factors for OS and PFS were identified using univariate regression models. Variables with a P<0.1 in univariate analysis were included in the multivariate regression model to correct for the interference of confounding factors to identify the independent prognostic factors of OS and PFS. BCLC stage, BMI (kg/m^2^), tumor size, AFP >= 200 (ug/L), Hepatectomy before TACE, metformin use, and treatment after TACE were associated with OS in the univariate analysis (P<0.1). And BCLC stage, tumor size, Hepatectomy before TACE, and metformin use were the prognosis factors for PFS (P<0.1), as shown in **Table 2**.

**Table 2:** Univariate and multivariate competing risk regression analysis for Overall survival and recurrence in the whole cohort.

Characteristics	Overall survival				Recurrence			
	Univariate analysis		Multivariate analysis‡		Univariate analysis		Multivariate analysis‡	
	HR (95% CI)	P-value	HR (95% CI)	P-value	HR (95% CI)	P-value	HR (95% CI)	P-value
Age (years)								
<60	1				1			
>60	0.95(0.56-1.6)	0.837			0.8(0.53-1.22)	0.302		
Gender								
Female	1				1			
Male	0.7(0.35-1.4)	0.316			0.98(0.52-1.85)	0.948		
BCLC stage								
A	1				1			
B	2.63(1.3-5.33)	0.007	2.59(1.23 - 5.44)	0.0124	1.23(0.76-2.01)	0.399	1.15(0.69 - 1.94)	0.5892
C	6.7(3.25-13.82)	0	9.17(3.81 - 22.1)	0	1.76(1.03-3)	0.039	1.57(0.87 - 2.82)	0.1338
BMI (kg/m2)								
<24	1				1			
24-27.9	0.41(0.23-0.74)	0.003	0.34(0.18 - 0.64)	0.0009	0.76(0.45-1.3)	0.324		
≥28	0.46(0.22-0.96)	0.039	0.28(0.13 - 0.62)	0.0018	0.76(0.41-1.43)	0.4		
Child-Pugh Score								
A	1				1			
B	1.47(0.72-3.01)	0.29			1.46(0.75-2.86)	0.264		
Tumor number								
single	1				1			
multiple	1.42(0.79-2.54)	0.237			0.77(0.5-1.18)	0.228		
Tumor size (cm)								
<=5	1				1			
>5	1.88(1.1-3.23)	0.022	1.51(0.83 - 2.76)	0.1747	1.43(0.94-2.19)	0.097	1.16(0.73 - 1.84)	0.5289
AFP (ug/L)								
<200	1				1			
>=200	1.84(1.09-3.12)	0.024	1.1(0.58 - 2.1)	0.7669	1.05(0.68-1.62)	0.842		
HBsAg (IU/ml)								
<1000	1				1			
>=1000	1.5(0.87-2.57)	0.141			1.14(0.75-1.74)	0.53		
Hepatectomy before TACE								
No	1				1			
Yes	0.14(0.02-1.01)	0.051	0.25(0.03 - 1.9)	0.1802	0.4(0.17-0.93)	0.033	0.71(0.29 - 1.74)	0.4507
Metformin use								
No	1				1			
Yes	0.59(0.35-1.01)	0.057	0.45(0.24 - 0.83)	0.0107	0.49(0.31-0.76)	0.002	0.52(0.31 - 0.85)	0.0091
Sorafenib after TACE								
No	1				1			
Yes	1.41(0.83-2.41)	0.205			1.04(0.68-1.61)	0.846		
Treatment after TACE†								
No	1				1			
Yes	0.48(0.22-1.01)	0.053	0.94(0.39 - 2.26)	0.8839	0.82(0.49-1.38)	0.458		

BCLC, Barcelona Clinic Liver Cancer; BMI, body mass index; ECOG, Eastern Cooperative Oncology Group; FBG Fasting blood sugar; AFP, Alpha-fetoprotein; HBsAg, hepatitis B surface antigen; TACE, transarterial chemoembolization; HR, hazard ratio; 95% CI, 95% confidence interval.

†Treatment after TACE including hepatectomy, radiofrequency ablation and microwave ablation

‡:Cox proportional hazards model (risk factors with a P-value <= 0.1 in the Univariate analysis were included in multivariate analysis to identify the independent predictors of OS and PFS).

In the multivariate analysis, BCLC stage, BMI, and metformin use remained independent predictors for OS. Compared to patients with BCLC stage A, patients with BCLC stage B (HR 2.59, 95%CI 1.23-5.44, P=0.0124) and stage C (HR 9.17, 95%CI 3.81-22.10, P<0.0001) were significantly associated with a poorer outcome. And patients with higher BMI (BMI 24-27.9 kg/m2 HR 0.34, 95% CI 0.18-0.64, P<0.0001; BMI >=28 kg/m2 HR 0.28, 95% CI 0.13-0.62, P=0.0018). Notably, the use of metformin was not only associated with the superior OS (HR 0.45, 95% CI 0.24-0.83, p=0.0107) but also related to the longer PFS (HR 0.52, 95%CI 0.31-0.85, p=0.0091). We further performed the duration-response analysis for 50 metformin users based on the different cumulative duration that patients took. Our results showed that the longer duration of metformin use was not associated with a lower risk of death as well as a lower recurrence after adjusting for BCLC stage and BMI level, as shown in **Table S1**.

### Sub-analysis based on BCLC staging system: overall survival and progression-free survival

A subgroup analysis based on the BCLC staging system was performed to investigate the impact of metformin in patients with different BCLC stages. The clinical characteristics of the patients as shown in **Table S2**. A total of 43 patients were diagnosed with BCLC stage A, 39 with BCLC stage B, and 41 with BCLC stage C.

**Figure 2:**
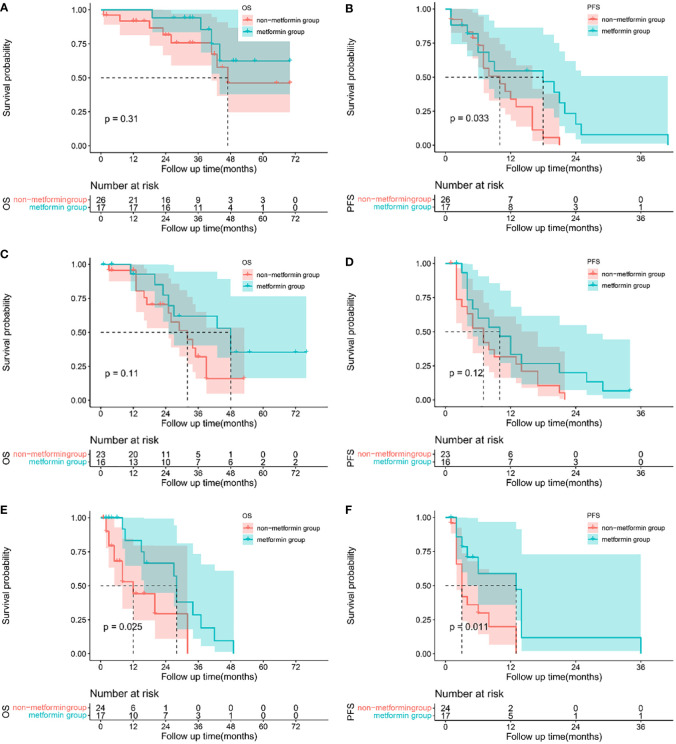
Sub-group analysis: Kaplan-Meier plots comparing metformin users versus non-metformin users among HCC patients with T2DM undergoing TACE based on BCLC staging system. Overall survival curves of patients with BCLC stage A **(A)**, stage B **(C)** and stage C **(E)**, and progression-free survival curves of patients for BCLC stage A**(B)**, stage B **(D)** and stage C**(F)**.

When stratified by the BCLC staging system, metformin increased the 1-, 2- and 3-year OS in patients with BCLC stage A (1-year 100% vs 91.80%, 2-year 100% vs 78.95%, 3-year 100% vs 72.87%, P = 0.313), **Figure 2A**. For patients with BCLC stage B, patients treated with metformin had a better OS (1-year 92.86% vs 95.65%, 2-year 77.38% vs 70.48% and 3-year 61.9% vs 32.04%, P=0.105), **Figure 2C**. Metformin was associated with the significant increased 1, 2 and 3-year OS in patients with BCLC stage C (1-year 83.33% vs 44.12%, 2-year 66.67% vs 29.41%, 3-year 28.57% vs 0.00%, P=0.025), **Figure 2E**. The median OS between patients treated with metformin and other antidiabetics in different BCLC stages was as follows, stage B 48 months (95% CI 26.442-69.558) vs 32 months (95% CI 20.900-43.100), stage C 28 months (95% CI 26.448-29.512) vs 12 months (95% CI 4.402-19.598), respectively. We didn’t obtain the median OS of patients with BCLC stage A due to the lack of death events. In terms of PFS, the use of metformin was associated with a reductive risk of recurrence than other antidiabetics in the each of BCLC stage (stage A 1-year 54.62% vs 33.88%, 2-year 15.61% vs 0.00%, 3-year 7.80% vs 0.00%, p=0.033; stage B 1-year 46.67% vs 31.58%, 2-year 20.00% vs 0.00%, 3-year 0% vs 0.00% P=0.124; stage C 1-year 58.93% vs 19.97%, 2-year 11.79% vs 0.00%, 3-year 0.00% vs 0.00%, P=0.011, as shown in **Figures 2B, D, F**. The median PFS between patients treated with metformin and other antidiabetics in different BCLC stages were as follows: stage A 18 months (95% CI 4.220-31.780) vs 10 months (95% CI 4.686-15.314), stage B 10 months (95% CI 4.319-15.681) vs 7 months (95% CI 3.836-10.164), stage C 13 months (95% CI 7.708-18.292) vs 3 months (95% CI 2.017-3.983), respectively.

### Propensity score match analysis

As multiple tumors were more common in patients treated with metformin than those treated with other antidiabetics, a one-to-one PSM was applied including 123 patients in total to reduce selection bias. A propensity score was calculated for each patient using a logistic regression model with 3 variables, including tumor number, BMI level, and BCLC stage.

**Figure 3:**
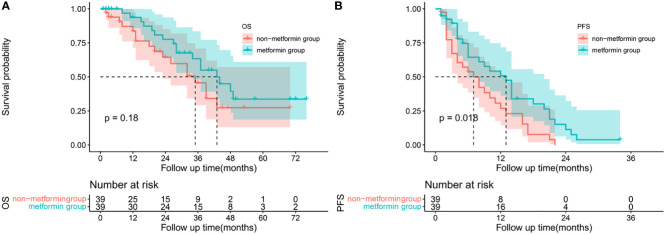
Survival analysis: Kaplan-Meier plots comparing metformin users versus non-metformin users among HCC patients with T2DM undergoing TACE. Overall survival **(A)** and progression-free survival **(B)** curves of matched population.

After matching, no statistically differences in any of the clinic characteristics were observed between the two groups, as shown in **Table 3**. Although without statistically insignificant, the metformin group had a higher proportion of ORR (66.67% vs 64.10%, p= 1.0000) and DCR (97.44% vs 92.31%, p=0.6077) compared with the non-metformin group. Patients treated with metformin had a better OS (1-year 93.75% vs 83.61%, 2-year 77.59% vs 64.61%, 3-year 63.37% vs 45.55%) and PFS (1-year 50.86% vs 26.88%, 2-year % 11.3 vs 0.00%, 3-year 0.00% vs 0.00%). The use of metformin was associated with a numerically longer m OS of 43 months (95% CI 32.498-53.502) than the use of other anti-diabetics of 35 months (95% CI 24.787-45.213), but the difference did not reach statistical significance(P=0.183), **Figure 3A**. Similarly, patients in the metformin group had a significantly longer m PFS of 13 months (95% CI 9.295-16.705) compared to the non-metformin group of 7 months (95%CI 3.353-10.647) (p=0.018), as shown in **Figure 3B**.

**Table 3:** Clinical characteristics between the metformin group and the non-metformin group of matched population.

Characteristic	Overall	Non-metformin Group	Metformin Group	P-Value
	n=78	n=39	n=39	
Age (%)				0.6435
<=60 years	47 (60.26)	25 (64.10)	22 (56.41)	
>60 years	31 (39.74)	14 (35.90)	17 (43.59)	
Gender (%)				1
Female	11 (14.10)	6 (15.38)	5 (12.82)	
Male	67 (85.90)	33 (84.62)	34 (87.18)	
Etiology (%)				0.6626
HBV	60 (76.92)	28 (71.79)	32 (82.05)	
HCV	5 (6.41)	4 (10.26)	1 (2.56)	
NAFLD	2 (2.56)	1 (2.56)	1 (2.56)	
Alcohol	3 (3.85)	2 (5.13)	1 (2.56)	
others	8 (10.26)	4 (10.26)	4 (10.26)	
BMI (%)				1
<24 kg/m2	18 (23.08)	9 (23.08)	9 (23.08)	
24-27.9 kg/m2	42 (53.85)	21 (53.85)	21 (53.85)	
≥28 kg/m2	18 (23.08)	9 (23.08)	9 (23.08)	
Child-Pugh Score (%)				1
A	69 (88.46)	35 (89.74)	34 (87.18)	
B	9 (11.54)	4 (10.26)	5 (12.82)	
BCLC stage (%)				1
A	26 (33.33)	13 (33.33)	13 (33.33)	
B	26 (33.33)	13 (33.33)	13 (33.33)	
C	26 (33.33)	13 (33.33)	13 (33.33)	
Liver cirrhosis (%)				0.2726
Absent	17 (21.79)	11 (28.21)	6 (15.38)	
Present	61 (78.21)	28 (71.79)	33 (84.62)	
Size (%)				1
<=5 cm	28 (35.90)	14 (35.90)	14 (35.90)	
>5 cm	50 (64.10)	25 (64.10)	25 (64.10)	
Tumor number (%)				1
single	26 (33.33)	13 (33.33)	13 (33.33)	
multiple	52 (66.67)	26 (66.67)	26 (66.67)	
FBG (%)				0.5388
<7 mmol/L	18 (23.08)	11 (28.21)	7 (17.95)	
7.0-7.6 mmol/L	14 (17.95)	7 (17.95)	7 (17.95)	
≥7.7 mmol/L	46 (58.97)	21 (53.85)	25 (64.10)	
T2DM duration (months) †	55.500 [20.250, 120.250]	58.000 [16.000, 115.000]	53.000 [29.000, 118.500]	0.8337
Hepatectomy before TACE (%)	4 (5.13)	1 (2.56)	3 (7.69)	0.6077
ALT (U/L)	35.000 [23.050, 53.350]	35.500 [24.550, 52.700]	34.500 [22.200, 53.300]	0.9085
AST (U/L)	37.450 [27.875, 57.275]	39.000 [31.300, 54.900]	37.100 [24.200, 59.100]	0.8299
Times of TACE	3.500 [2.000, 7.000]	3.000 [2.000, 6.500]	4.000 [2.500, 7.000]	0.6646
Sorafenib after TACE (%)	58 (74.36)	32 (82.05)	26 (66.67)	0.1948
Treatment after TACE‡ (%)	66 (84.62)	33 (84.62)	33 (84.62)	1.0000
Tumor response (%)				0.4025
CR	5 (6.41)	1 (2.56)	4 (10.26)	
PR	46 (58.97)	24 (61.54)	22 (56.41)	
SD	23 (29.49)	11 (28.21)	12 (30.77)	
PD	4 (5.13)	3 (7.69)	1 (2.56)	
ORR (%) §	51 (65.38)	25 (64.10)	26 (66.67)	1.0000
DCR (%) ¶	74 (94.87)	36 (92.31)	38 (97.44)	0.6077

Data are shown as numbers of events with percentages in parentheses or median [interquartile range, IQR]. HBV, hepatitis B virus; HCV, hepatitis C virus; NAFLD, nonalcoholic fatty liver disease; BMI, body mass index; ECOG, Eastern Cooperative Oncology Group; BCLC, Barcelona Clinic Liver Cancer; T2DM, type 2 diabetic mellitus; FBG, Fasting blood sugar; ALT, Alanine aminotransferase; AST, Aspartate aminotransferase; TACE, transarterial chemoembolization; CR, complete response; PR, partial response; SD, stable disease; PD, progressive disease; ORR, Objective Response Rate; DCR, disease control rate

†: T2DM duration before the first TACE

‡: Including radiofrequency, microwave ablation and Hepatectomy.

§: Sum of CR and PR.

¶: Sum of CR, PR, and SD.

## Discussion

TACE is the first-line treatment modality for BCLC stage B HCC according to the BCLC staging system (3). However, 96.2% of the patients had tumor recurrence whin 9 months (13). Early recurrence is still the main reason for poor long-term survival after TACE. It is vital to prevent recurrence after TACE in improving the long-term survival of patients. Metformin has recently attracted increasing attention due to its effects on inhibiting tumor cell proliferation and promoting tumor cell apoptosis (14). Some studies reported controversial data on its antitumor effects in non-surgical treatment (15, 16). As far as we know, this is the first study to demonstrate the potential of the combination of metformin and TACE in HCC patients with T2DM.

T2DM is significantly associated with poor prognosis for many cancers, including liver cancer, endometrial cancer, breast cancer, pancreatic cancer, and colorectal cancer (17). Obesity is a major determinant of insulin resistance and the development of T2DM, and only 10 to 25% of obese adults remain metabolically healthy and insulin-sensitive (18). In our study, almost 69.57% of all patients had overweight, which was in line with recent studies. Hyperglycemia, insulin resistance, hyperinsulinemia, and obesity all play important roles in cytokine production, inflammatory responses, and oxidative stress, which results in a pro-carcinogenic environment (19). It is reported that the development of insulin resistance and subsequent T2DM not only increased the incidence of HCC but also increased the risk of tumor recurrence in HCC patients who received TACE (20).

Metformin is recommended first-line treatment for patients with T2DM. It can reduce the production of hepatic glucose, thus indirectly reducing circulating glucose levels and mitigating the tumor-promoting effects of hyperglycemia. In our study, we found that metformin significantly increased both OS and PFS in HCC patients with T2DM. Importantly, metformin remained the protective factor for OS and PFS after adjusting for BCLC stage, BMI, tumor size, and other confounders. It is worth mentioning that the metformin group still surpassed the non-metformin group in OS and PFS after matching. And the metformin had a better ORR and DCR compared with the non-metformin group in either the matched population or the unmatched population. The insignificant P value could be due to the small number of patients. However, the risk of tumors seemed to be increased for patients with other antidiabetics. In the past decade, increasing evidence has shown a higher risk of HCC incidence in diabetic patients treated with insulin or Sulfonylureas (an insulin secretagogue) (21).

The mammalian target of rapamycin (mTOR) plays an important role in the anti-HCC effects of metformin. The upregulation of mTOR is associated with lower tumor differentiation, worse prognosis, and early recurrence (22). Metformin can effectively inhibit HCC growth by inhibiting mTOR through the phosphatidylinositol-3-kinase (PI3K)/protein kinase B(AKT)/mTOR signal pathway (14). Metformin also has antitumor effects by reducing mitochondrial complex I in the respiratory chain to activate AMPK (23). The mechanisms of action of metformin were complex and further research is required to fully comprehend how metformin works in its target population. Besides, it is reported metformin inhibited the proliferation of hepatoma cell lines in a dose-dependent manner (24). And Chen’s study suggested that each incremental year increase in metformin use resulted in a 7% reduction in the risk of HCC in diabetic patients and metformin (25). Similarly, Chan et al. found that preoperative usage of metformin would reduce the risk of death after liver resection in a dose- and duration-dependent manner (26). However, there was no duration-response relationship between the use of metformin and the risk of post-TACE death or tumor recurrence in our study. Notably, the information on treatment was obtained through prescriptions contained in the medical records of patients. Therefore, a gap between prescribed and actual doses could bias the results. Some individuals using metformin may have become “non-users” because of treatment interruption. Thus, misclassification of exposure occurred. To sum up, large and randomized trials in well-selected patients treated with different dosages are warranted to confirm the value of metformin in HCC recurrence.

TACE is the most common treatment for unresectable HCC. In clinical practice, TACE is widely used for different BCLC stages of HCC (BCLC stage A or even BCLC stage C), and almost half of TACE treatments are performed in patients with BCLC stage C (27, 28). Jin et al. (29)compared the outcomes of liver resection and TACE for solitary large HCC. They reported that liver resection offered a significantly better 5-year survival rate in the surgical group than in the TACE group (65% vs 17%, P < 0.01) irrespective of tumor size. And the time to progression was lower in patients who underwent the TACE. Disease progression was the most common cause of death for these patients. Chan et al. reported that T2DM was associated with high recurrence and poor long-term survival. Notably, metformin use was associated with lower recurrence and a better 5-year OS in the early HCC patients undergoing RFA (30). Similar results were presented in the patients with BCLC stage 0 or A undergoing liver resection (26). However, these studies have focused on radical treatment, including RFA and liver resection. We compared the OS and PFS between the metformin and non-metformin group in HCC patients with T2DM undergoing TACE. And we found that the combination of metformin and TACE was associated with a better survival outcome. Subsequently, the sub-analysis was performed to investigate whether metformin could give a survival advantage in each BCLC stage of HCC. Our results show that the 3-year survival rate of patients in BCLC stages A to C who were treated with metformin all showed a trend of improvement.

The limitation of our study was the retrospective nature and based on a single center. Our study comprised a limited number of people, the majority of whom had previously been infected with HBV. Our results support the benefits of metformin in patients with HCC undergoing TACE. Additionally, some patients used sorafenib in our study. Recently, several studies revealed a relationship between metformin and sorafenib. Whether the combination of metformin and sorafenib is beneficial or harmful is still controversial (31, 32). We did not do additional subgroup analysis based on sorafenib because of the small number of patients in our study. But we are conducting a multicenter retrospective study focused on the interaction of metformin and sorafenib. Finally, further prospective research is needed to determine that metformin has the same effect on HCC patients who do not have diabetes.

In conclusion, metformin increased long-term survival time and significantly prevented recurrence after TACE in HCC patients with T2DM. Our results suggested that patients with BCLC stage A/B/C may all benefit from metformin.

## Data availability statement

The datasets presented in this study can be found in online repositories. The names of the repository/repositories and accession number(s) can be found in the article/**Supplementary Material**.

## Author contributions

Topic selection and guidance: J-JH; reviewed cases, made statistical analysis and wrote the manuscripts, M-LC; Acquisition and analysis of data: C-XW, HZ; Critical revision of the manuscript: C-XW, HZ, and Y-DS. All author contributed to the article and approved the submitted version. All authors contributed to the article and approved the submitted version.

## Funding

This item was supported by the National Key Research and Development Program (No.2018YFE0126500), the Natural Science Foundation of Shandong Province (No. ZR2021MH060), Plan for Science and Technology Development of Ji’nan (No.201907124) and the Start-up fund of Shandong Cancer Hospital (2020-PYB20). The funding bodies played no role in the design of the study and collection, analysis, and interpretation of data and in writing the manuscript.

## Acknowledgments

The authors acknowledge partial support of the National key & D Program “International innovation cooperation among governments” special projects (grant number 2018YFE0126500), Natural Science Foundation of Heilongjiang Province of China (grant number ZR202103030646), Plan for Science and Technology Development of Ji’nan (grant number 201907124), and Start-up fund of Shandong Cancer Hospital (2020-PYB20).

## Conflict of interest

The authors declare that the research was conducted in the absence of any commercial or financial relationships that could be construed as a potential conflict of interest.

## Publisher’s note

All claims expressed in this article are solely those of the authors and do not necessarily represent those of their affiliated organizations, or those of the publisher, the editors and the reviewers. Any product that may be evaluated in this article, or claim that may be made by its manufacturer, is not guaranteed or endorsed by the publisher.
