# Supplementary material for: Transarterial chemoembolization combined with metformin improves the prognosis of hepatocellular carcinoma patients with type 2 diabetes
Source: Front Endocrinol (Lausanne). 2022 Sep 15;13:996228. doi: 10.3389/fendo.2022.996228 (PMC9520252; doi:10.3389/fendo.2022.996228)
Supplement: Supplementary file 1 [file Table_1.docx]

Table S1. Univariate and multivariate competing risk regression analysis based on the duration of metformin use for overall survival and progression.

|  | Overall survival | | | | Progression | | | |
| --- | --- | --- | --- | --- | --- | --- | --- | --- |
| Duration of metformin† | Univariate analysis | | Multivariate analysis‡ | | Univariate analysis | | Multivariate analysis‡ | |
|  | HR (95% CI) | P-value | HR (95% CI) | P-value | HR (95% CI) | P-value | HR (95% CI) | P-value |
| <=36 months | 1 |  | 1 |  | 1 |  | 1 |  |
| 36-72 moths | 1.6(0.51-5.01) | 0.419 | 5.94(1.23 - 28.66) | 0.0266 | 0.85(0.31-2.33) | 0.753 | 0.91(0.31 - 2.69) | 0.8682 |
| > 72 months | 1.19(0.36-3.92) | 0.773 | 3.1(0.8 - 12.07) | 0.1027 | 1.03(0.45-2.37) | 0.949 | 0.99(0.39 - 2.5) | 0.9823 |

HR, hazard ratio; 95 % CI, 95% confidence interval;

†: The duration of metformin before the first TACE;

‡: Cox proportional hazards model (Adjusting for BCLC stage, BMI level).
