# Supplementary material for: Transarterial chemoembolization combined with metformin improves the prognosis of hepatocellular carcinoma patients with type 2 diabetes
Source: Front Endocrinol (Lausanne). 2022 Sep 15;13:996228. doi: 10.3389/fendo.2022.996228 (PMC9520252; doi:10.3389/fendo.2022.996228)
Supplement: Supplementary file 2 [file Table_2.docx]

Table S2. Clinical characteristics between the metformin group and the non-metformin group in HCC patients undergoing TACE based on BCLC staging system in the whole cohort.

| Characteristics | BCLC A | | | BCLC B | | | BCLC C | | |
| --- | --- | --- | --- | --- | --- | --- | --- | --- | --- |
|  | Non-metformin | metformin | P-Value | Non-metformin | metformin | P-Value | Non-metformin | metformin | P-Value |
|  | N=26 | N=17 |  | N=23 | N=16 |  | N=24 | N=17 |  |
| Age (%) |  |  | 1.0000 |  |  | 0.2333 |  |  | 0.6926 |
| <=60 yr | 15 (57.69) | 9 (52.94) |  | 17 (73.91) | 8 (50.00) |  | 14 (58.33) | 8 (47.06) |  |
| >60 yr | 11 (42.31) | 8 (47.06) |  | 6 (26.09) | 8 (50.00) |  | 10 (41.67) | 9 (52.94) |  |
| Gender (%) |  |  | 0.9304 |  |  | 0.3720 |  |  | 0.3440 |
| Female | 3 (11.54) | 1 (5.88) |  | 3 (13.04) | 0 (0.00) |  | 3 (12.50) | 5 (29.41) |  |
| Male | 23 (88.46) | 16 (94.12) |  | 20 (86.96) | 16 (100.00) |  | 21 (87.50) | 12 (70.59) |  |
| Etiology (%) |  |  | 0.6606 |  |  | 0.4154 |  |  | 0.2717 |
| HBV | 23 (88.46) | 14 (82.35) |  | 17 (73.91) | 14 (87.50) |  | 17 (70.83) | 13 (76.47) |  |
| HCV | 1 (3.85) | 1 (5.88) |  | 2 (8.70) | 0 (0.00) |  | 2 (8.33) | 1 (5.88) |  |
| NAFLD | 1 (3.85) | 0 (0.00) |  | 0 (0.00) | 0 (0.00) |  | 0 (0.00) | 1 (5.88) |  |
| Alcohol | 1 (3.85) | 1 (5.88) |  | 0 (0.00) | 0 (0.00) |  | 4 (16.67) | 0 (0.00) |  |
| other | 0 (0.00) | 1 (5.88) |  | 4 (17.39) | 2 (12.50) |  | 1 (4.17) | 2 (11.76) |  |
| BMI (%) |  |  | 0.9604 |  |  | 0.1971 |  |  | 0.2441 |
| ＜24 kg/m^2^ | 7 (26.92) | 4 (23.53) |  | 7 (30.43) | 3 (18.75) |  | 10 (41.67) | 3 (17.65) |  |
| 24-27.9 kg/m^2^ | 15 (57.69) | 10 (58.82) |  | 13 (56.52) | 7 (43.75) |  | 10 (41.67) | 9 (52.94) |  |
| ≥28 kg/m^2^ | 4 (15.38) | 3 (17.65) |  | 3 (13.04) | 6 (37.50) |  | 4 (16.67) | 5 (29.41) |  |
| Child-Pugh Score (%) |  |  | 0.4009 |  |  | 1.0000 |  |  | 0.6147 |
| A | 23 (88.46) | 17 (100.00) |  | 19 (82.61) | 14 (87.50) |  | 21 (87.50) | 13 (76.47) |  |
| B | 3 (11.54) | 0 (0.00) |  | 4 (17.39) | 2 (12.50) |  | 3 (12.50) | 4 (23.53) |  |
| Liver cirrhosis (%) |  |  | 0.3100 |  |  | 0.2322 |  |  | 0.2243 |
| Absent | 2 (7.69) | 4 (23.53) |  | 8 (34.78) | 2 (12.50) |  | 8 (33.33) | 2 (11.76) |  |
| Present | 24 (92.31) | 13 (76.47) |  | 15 (65.22) | 14 (87.50) |  | 16 (66.67) | 15 (88.24) |  |
| Size (%) |  |  | 1.0000 |  |  | 1.0000 |  |  | 0.5017 |
| <=5 cm | 16 (61.54) | 10 (58.82) |  | 8 (34.78) | 6 (37.50) |  | 5 (20.83) | 6 (35.29) |  |
| >5 cm | 10 (38.46) | 7 (41.18) |  | 15 (65.22) | 10 (62.50) |  | 19 (79.17) | 11 (64.71) |  |
| Tumor number (%) |  |  | 0.2221 |  |  | - |  |  | 0.0584 |
| single | 21 (80.77) | 10 (58.82) |  | 0 (0.00) | 0 (0.00) |  | 14 (58.33) | 4 (23.53) |  |
| multiple | 5 (19.23) | 7 (41.18) |  | 23 (100.00) | 16 (100.00) |  | 10 (41.67) | 13 (76.47) |  |
| FBG (%) |  |  | 0.1735 |  |  | 0.7080 |  |  | 0.5069 |
| ＜7 mmol/L | 7 (26.92) | 1 (5.88) |  | 7 (30.43) | 3 (18.75) |  | 9 (37.50) | 4 (23.53) |  |
| 7.0-7.6 mmol/L | 2 (7.69) | 3 (17.65) |  | 4 (17.39) | 3 (18.75) |  | 2 (8.33) | 3 (17.65) |  |
| ≥7.7 mmol/L | 17 (65.38) | 13 (76.47) |  | 12 (52.17) | 10 (62.50) |  | 13 (54.17) | 10 (58.82) |  |
| T2DM duration (months) † | 51.000 [20.250, 88.000] | 48.000 [13.000, 131.000] | 0.6457 | 71.000 [23.500, 128.000] | 61.500 [33.500, 101.750] | 0.7211 | 60.500 [12.000, 126.000] | 44.000 [36.000, 127.000] | 0.7207 |
| Hepatectomy before TACE (%) | 1 (3.85) | 1 (5.88) | 1.0000 | 1 (4.35) | 0 (0.00) | 1.0000 | 0 (0.00) | 0 (0.00) | - |
| ALT (U/L) | 30.500 [21.325, 49.775] | 23.000 [17.500, 38.900] | 0.2968 | 34.100 [20.000, 55.650] | 35.500 [27.425, 68.500] | 0.4157 | 27.350 [18.650, 50.325] | 35.500 [21.800, 58.000] | 0.3683 |
| AST (U/L) | 33.100 [23.575, 48.100] | 24.400 [21.000, 37.000] | 0.1090 | 34.600 [24.100, 55.950] | 43.750 [23.500, 53.925] | 0.6894 | 40.650 [33.300, 58.850] | 51.500 [33.500, 67.400] | 0.6624 |
| Times of TACE | 4.000 [2.250, 8.750] | 4.000 [2.000, 8.000] | 0.7079 | 6.000 [2.500, 9.500] | 5.000 [3.000, 7.000] | 0.4220 | 3.000 [2.000, 4.250] | 3.000 [2.000, 7.000] | 0.3611 |
| Sorafenib after TACE (%) | 6 (23.08) | 2 (11.76) | 0.5953 | 12 (52.17) | 7 (43.75) | 0.8477 | 4 (16.67) | 7 (41.18) | 0.1653 |
| Treatment after TACE‡ (%) | 7 (26.92) | 6 (35.29) | 0.8066 | 4 (17.39) | 2 (12.50) | 0.8066 | 0 (0.00) | 1 (5.88) | 0.8607 |
| Tumor response (%) |  |  | 0.5671 |  |  | 0.3055 |  |  | 0.8295 |
| CR | 4 (15.38) | 2 (11.76) |  | 0 (0.00) | 1 (6.25) |  | 1 (4.17) | 1 (5.88) |  |
| PR | 14 (53.85) | 12 (70.59) |  | 14 (60.87) | 11 (68.75) |  | 10 (41.67) | 9 (52.94) |  |
| SD | 6 (23.08) | 3 (17.65) |  | 6 (26.09) | 4 (25.00) |  | 10 (41.67) | 6 (35.29) |  |
| PD | 2 (7.69) | 0 (0.00) |  | 3 (13.04) | 0 (0.00) |  | 3 (12.50) | 1 (5.88) |  |
| ORR (%) § | 18 (69.23) | 14 (82.35) | 0.5440 | 14 (60.87) | 12 (75.00) | 0.5650 | 11 (45.83) | 10 (58.82) | 0.6152 |
| DCR (%) ¶ | 24 (92.31) | 17 (100.00) | 0.6668 | 20 (86.96) | 16 (100.00) | 0.3720 | 21 (87.50) | 16 (94.12) | 0.8655 |

Data are shown as numbers of events with percentages in parentheses or median [interquartile range, IQR]; HBV, hepatitis B virus; HCV, hepatitis C virus; NAFLD, nonalcoholic fatty liver disease; BMI, body mass index; ECOG, Eastern Cooperative Oncology Group; BCLC, Barcelona Clinic Liver Cancer; T2DM, type 2 diabetic mellitus; FBG Fasting blood sugar; ALT Alanine aminotransferase; AST, Aspartate aminotransferase; TACE, transarterial chemoembolization; CR, complete response; PR, partial response; SD, stable disease; PD, progressive disease; ORR, Objective Response Rate; DCR, disease control rate

†:T2DM duration before TACE;

‡:Including radiofrequency and microwave ablation.

§: Sum of CR and PR.

¶: Sum of CR, PR, and SD.
